# The Path of Bronchiolitis Towards Intensive Care: Risk Factor Analysis in a Large Italian Cohort

**DOI:** 10.3390/jcm14155420

**Published:** 2025-08-01

**Authors:** Marco Maglione, Luca Pierri, Fabio Savoia, Camilla Calì, Roberta Ragucci, Marco Sarno, Giulia Ranucci, Emma Coppola, Francesco Nunziata, Antonino Di Toro, Vincenzo Tipo, Antonietta Giannattasio, the BRAND Study Group

**Affiliations:** 1 Pediatric Emergency Unit, Santobono-Pausilipon Children’s Hospital, 80129 Naples, Italy; ro.ragucci@studenti.unina.it (R.R.); v.tipo@santobonopausilipon.it (V.T.); a.giannattasio@santobonopausilipon.it (A.G.); 2 Neonatal Intensive Care Unit, Santobono-Pausilipon Children’s Hospital, 80129 Naples, Italy; l.pierri@santobonopausilipon.it (L.P.); a.ditoro@santobonopausilipon.it (A.D.T.); 3 Epidemiology, Biostatistics and Childhood Cancer Registry Unit, Santobono-Pausilipon Children’s Hospital, 80129 Naples, Italy; f.savoia@santobonopausilipon.it (F.S.); c.cali@santobonopausilipon.it (C.C.); 4 Chronic and Multifactorial Diseases Unit, Santobono-Pausilipon Children’s Hospital, 80129 Naples, Italy; m.sarno@santobonopausilipon.it; 5 Department of Pediatrics, AORN Sant’Anna E San Sebastiano, 81100 Caserta, Italy; giulia.ranucci@aorncaserta.it; 6 Department of Pediatrics, San Leonardo Hospital, 80053 Castellammare di Stabia, Italy; em.coppola@aslnapoli3-sud.it; 7 Pediatric Immunoreumatology Unit, Santobono-Pausilipon Children’s Hospital, 80129 Naples, Italy; f.nunziata@santobonopausilipon.it

**Keywords:** bronchiolitis, intensive care, risk factors

## Abstract

**Background/Objectives:** Bronchiolitis is the leading cause of hospitalization in infants under 12 months. While often self-limiting, a subset of cases evolves into severe disease requiring intensive care. This study aimed to identify risk factors for severe bronchiolitis in two consecutive respiratory syncytial virus (RSV) seasons (before and after the introduction of nirsevimab) in Southern Italy. **Methods:** A retrospective, multicenter cohort study was conducted on all infants ≤12 months hospitalized with bronchiolitis from October 2023 to March 2025. Patients were categorized by disease severity: those requiring Sub-Intensive or Intensive Care (IC group) and others (n-IC group). Demographic and clinical data, virological testing, and therapeutic interventions were analyzed. Multivariable logistic regression was used to identify independent risk factors for severe disease. **Results:** Among 1056 hospitalized infants, 10.5% required intensive care. RSV was detected in 73.5% of tested patients and was significantly associated with severe outcomes. Independent risk factors for IC admission included younger age (<3 months), comorbidities, and RSV infection. A 33% reduction in bronchiolitis admissions was observed in the second season (post-nirsevimab), although the rate of severe cases remained stable (about 10% in both seasons). **Conclusions:** Younger age, comorbidities, and RSV infection are significant predictors of severe bronchiolitis. Although overall admissions decreased post-nirsevimab, severe cases persisted. These findings underscore the need for targeted preventive strategies and highlight the potential role of intermediate care approaches in managing bronchiolitis severity.

## 1. Introduction

Bronchiolitis is a common disease affecting the lower (smaller) respiratory airways in infants (younger than 24 months of age). Typically caused by a viral infection, it results in breathing difficulties and can lead to poor feeding. The severity of bronchiolitis is variable. It normally has a mild course, but several risk factors, including preterm birth and chronic conditions, e.g., bronchopulmonary dysplasia or heart disease, have been associated with the development of severe illness [1,2]. Nevertheless, a noteworthy proportion of severe bronchiolitis is observed in otherwise healthy, full-term infants [3]. Although acute bronchiolitis has been well-known for decades, its therapeutic approach has remained largely unchanged and is essentially limited to respiratory and metabolic support [4]. In this context, high-flow nasal cannula (HFNC) systems have become widely accepted as respiratory support modalities for oxygen therapy in critically ill patients of all ages [4]. HFNC systems offer several advantages over low-flow oxygen in infants with bronchiolitis, including greater improvement in respiratory and heart rates, modest reduction in the length of hospital stay, and the shorter duration of oxygen therapy, with a decreased need for treatment escalation [4]. Nevertheless, although approximately 5% of hospitalized infants still experience severe bronchiolitis, requiring admission to the Pediatric Intensive Care Unit (PICU) [5], key gaps remain in our understanding of how poor clinical outcomes develop in these children.

Respiratory syncytial virus (RSV) is the leading cause of acute bronchiolitis in infants and, more broadly, of lower respiratory tract infections in children under 5 years of age [6,7]. The recent development of immunoprophylactic agents against RSV has given a new impetus to the research on its burden on children, particularly with regard to severe cases. In July 2023, the Food and Drug Administration approved the monoclonal antibody nirsevimab (Beyfortus) for RSV prophylaxis [8]. Shortly thereafter, the Advisory Committee on Immunization Practices and the American Academy of Pediatrics recommended that all infants <8 months of age entering their first RSV season should receive nirsevimab, as well as infants at increased risk for severe RSV-bronchiolitis during their second year of life [9]. In Italy, nirsevimab has been administered since October 2024, with varying modalities according to individual regional plans. In the Campania Region, it was introduced in November 2024 and offered in maternity wards to newborns from 11 November 2024 to 14 April 2025, as well as through an outpatient program for infants born between 1 August 2024, and 10 November 2024 [10].

The aim of this study was to describe the clinical course of a large sample of infants with acute bronchiolitis during two consecutive RSV seasons (before and after the introduction of nirsevimab in the Campania region) and to assess risk factors potentially associated with a severe disease course and adverse outcomes.

## 2. Materials and Methods

### 2.1. Study Design

We conducted a two-year multicenter, retrospective cohort study coordinated by the Santobono-Pausilipon Children’s Hospital. This is the largest tertiary care pediatric hospital of Southern Italy and is the reference center for pediatric intensive care in Campania region. It is equipped with a Neonatology Ward, a Pediatric Emergency Department (that manages patients with moderate acute respiratory diseases), a Pediatric Sub-Intensive Care Unit (for patients with severe conditions not requiring invasive ventilation, intensive monitoring, or cardiac support), a Neonatal Intensive Care Unit (NICU), and a PICU. The study included patients admitted to the Santobono-Pausilipon Children’s Hospital and to other pediatric centers taking part to the Bronchiolitis and RSV After nirsevimab Diffusion (BRAND) Study Group, namely, AORN Sant’Anna E San Sebastiano (Caserta); San Leonardo Hospital (Castellammare di Stabia, Naples); Santa Maria della Pietà Hospital (Nola, Naples); and Santa Maria delle Grazie Hospital (Pozzuoli, Naples).

All consecutive children aged ≤12 months hospitalized with a clinical diagnosis of bronchiolitis observed between 1 October 2023 and 31 March 2025 were considered eligible. Patients admitted between 1 October 2023 and 31 March 2024 were assigned to the Season 1 group (pre-nirsevimab season, S1), while those admitted between 1 October 2024 and 31 March 2025 were assigned to the Season 2 group (nirsevimab post-marketing period, S2). The medical records of each patient were reviewed, collecting demographic and clinical data. A particular focus was given to potential risk factors, such as prematurity and comorbidities.

### 2.2. Population

The diagnosis of bronchiolitis was based on the clinical history and physical examination [11,12]: coryzal prodromes lasting 1 to 3 days, followed by persistent cough and either tachypnea and/or increased respiratory effort (presenting as nasal flaring, grunting, use of accessory muscles or intercostal and/or subcostal chest wall retractions), low peripheral oxygen saturation (SpO_2_) levels, apnea, skin color changes, feeding difficulties, and lethargy [11,12].

Mild cases not requiring hospitalization were excluded. Infants were also excluded if their hospital admission was for causes unrelated to bronchiolitis or in case of proven bacterial co-infection. Patients with repeated hospitalizations for bronchiolitis more than 30 days apart were considered eligible for inclusion as separate cases.

The analyzed variables included age, sex, prematurity, comorbidities, respiratory coinfections, laboratory parameters at admission, chest imaging, date of hospitalization and discharge, treatments during hospitalization, admission to the ICU and length of ICU stay, need for and duration of oxygen therapy, HFNC, and non-invasive and invasive respiratory support. Underlying conditions identified at admission were classified as comorbidities. Prematurity was defined in case of gestational age of <37 weeks.

### 2.3. Outcomes

The primary outcome of the study was “severe disease,” defined based on the patient’s transition to a Sub-Intensive Care Unit and/or PICU or NICU. Severity was treated as a binary variable, and patients were categorized into two groups: Non-Intensive Care Group (n-IC group) and Intensive Care Group (IC group), based on the transfer to NICU, PICU or Sub-Intensive Care Unit.

Criteria for ICU admission were mainly based on respiratory support requirements and included the following: escalating respiratory needs beyond HFNC settings of 2 L/min/kg and FiO_2_ > 50% to maintain SpO_2_ ≥ 92%, need for non-invasive or invasive ventilation, and rapid clinical deterioration requiring ICU-level monitoring or requirement of vasoactive support.

The initiation of HFNC therapy alone was not considered equivalent to non-invasive ventilation, nor sufficient to assign the patient to the IC group, due to its widespread use in general pediatric wards [13].

### 2.4. Methods

Etiology of bronchiolitis was based on polymerase chain reaction testing or, in some cases of RSV infection, on rapid antigen test results from nasopharyngeal swabs collected at hospital admission or within 2 days after hospitalization. In most cases, patients underwent testing with an extensive respiratory panel. Respiratory pathogens were identified using the FilmArray Respiratory Panel (FilmArray^®^ Respiratory Panel, BioFire Diagnostics LLC, 390 Wakara Way, Salt Lake City, UT, USA) on nasopharyngeal swabs. This test detects Influenza A virus (FluA) (H1N1, H1N1 2009, and H3N2), Influenza B virus (FluB), RSV, human parainfluenza viruses 1–4 (PIVs 1–4), adenovirus (ADV), rhino/enteroviruses (HRV/EV), metapneumovirus (HMPV), human coronaviruses (HCoVs: 229E, HKU1, OC43, and NL63), Middle East Respiratory Syndrome virus (MERS), SARS-CoV-2, Bordetella pertussis (BORp), Bordetella parapertussis (BORpa), Chlamydia pneumoniae (CLAMP), and Mycoplasma pneumoniae (MYCOP). In other cases, a limited viral panel, including RSV, SARS-CoV-2, and Flu A and B, was performed (GeneXpert, Chepheid, 904 Caribbean Drive, Sunnyvale, CA 94089, USA). All tests were performed as part of normal clinical procedures.

Viral coinfection was defined as the simultaneous detection of more than one respiratory virus in a single patient sample, regardless of the specific viruses identified. RSV coinfection was defined as the presence of RSV along with one or more additional viruses. Viral coinfection was analyzed only in patients who underwent testing with the extensive respiratory panel.

### 2.5. Statistical Analysis

Categorical variables are shown as numbers and percentages, while continuous variables are shown as median and interquartile ranges (IQr). Categorical variables were evaluated with chi-square test, while Wilcoxon rank-sum test was used for continuous variables (Table 1). Univariate and multivariate logistic regression models were developed to assess independent variables associated with severity (n-IC group versus IC group). Patients were included in the multivariable logistic regression model only if all required variables were available. No data imputation was performed. The independent variables examined were age classes (<1 month, 1–2 months, 3–5 months, 6–12 months), comorbidities, RSV, C-reactive protein (CRP), and season of admission (Table 2). A two tailed *p*-value < 0.05 was considered statistically significant. Statistical analysis was performed using StataCorp LLC Stata 17.0 (College Station, TX, USA).

## 3. Results

### 3.1. Characteristics of the Study Population

A total of 1056 patients with bronchiolitis were included in the analysis. Of these, 111 (10.5%) met criteria for severe disease (IC group), while 945 (89.5%) belonged to the n-IC group. Characteristics of children with bronchiolitis between the IC and n-IC groups are summarized in Table 1.

The majority (n = 650, 61.5%) of patients were younger than 3 months. The most common symptom at presentation was respiratory distress (n = 821, 77.8%) followed by cough (n = 467, 44.2%). The same symptoms’ distribution was recorded within the n-IC group (respiratory distress: 76.9%; cough: 43.8%) and the IC group (respiratory distress: 86.6%; cough: 48.6%).

An extensive respiratory panel was performed in 552/1056 (52.3%) cases; 488 (46.2%) patients were tested for RSV only, while in 16 (1.5%) patients no respiratory panel was performed. RSV was the most detected virus, with a total of 765 (73.5%) RSV-positive patients out of 1040 tested for RSV. Even considering only the group of 552 patients who underwent the extensive respiratory panel, RSV was still the most common pathogen detected (354 RSV-positive specimens out of 552, 64.1%). In addition to RSV, 173/552 (31.3%) infants had a concurrent respiratory virus identified. The most common viruses detected as coinfecting agents were HCoVs (n = 22, 12.7%) and ADV (n = 15, 8.7%). In most coinfections two pathogens were detected; 24/173 (13.9%) patients had three or more coinfecting viruses. Interestingly, the proportion of patients undergoing an extensive respiratory panel was higher in the IC group than in the n-IC group (69.4% versus 50.3%, *p* = 0.001), suggesting a more intense diagnostic effort in severe cases. Nonetheless, despite this greater diagnostic pressure in the IC group, the proportion of coinfections was not significantly higher in these patients (Table 1).

Patients with severe bronchiolitis were younger, were more likely to have comorbidities, and had higher CRP values compared to the n-IC group (Table 1).

Oxygen therapy was administered to 646 (61.2%) patients with a median duration of 4 days (range 3–6), representing a period overlapping with the duration of hospital stay. Almost all (95.5%) patients from the IC group and 57.1% of n-IC group subjects received oxygen supplementation (*p* < 0.05). HFNC was the type of oxygen support administered in most cases (555/646, 85.9%), while 73 (11.3%) patients required low-flow oxygen supplementation. Eighteen (2.8%) children required other modalities of ventilation. As for other kinds of treatment, patients belonging to the IC group received more frequently antibiotics, steroids (mainly parenterally), and inhaled adrenaline. Abnormal chest X-ray (the detection of consolidation or infiltration) was also more common in the IC group (Table 1).

### 3.2. Comparison Between Two Seasons

**Figure 1:**
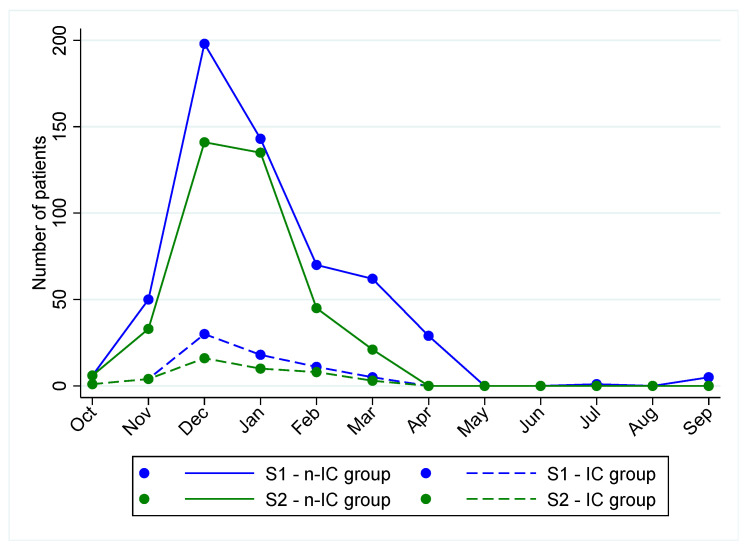
Distribution of admitted patients over the two bronchiolitis seasons (2023/2024: S1 and 2024/2025: S2, respectively) according to the need for Intensive Care (IC group and n-IC group, respectively).

The annual number of hospitalizations due to bronchiolitis and the proportion of severe infections were recorded. Six hundred and thirty-three (59.9%) infants were observed in the 2023–2024 season (S1) and 423 (40.1%) patients were included in the 2024–2025 season (S2) (Figure 1).

In both seasons, the peak of admissions for bronchiolitis was observed between the end of December and the first week of January. When we compared the total number of hospitalizations in the two subsequent seasons, we observed a decrease of 33% in S2 compared to S1. Similarly, RSV-related bronchiolitis decreased by an absolute number in S2 (n = 326) compared to S1 (n = 439). In addition, the absolute number of patients included in the IC group decreased in S2 (n = 42) compared to S1 (n = 69). Nevertheless, the rate of admission to sub-intensive or intensive care remained unchanged between the two periods (S2: 10.9% versus S1: 9.9%, *p* > 0.05) (Figure 1). Notably, during S2, 8 out of 42 IC patients (19%) and 79 out of 381 n-IC patients (20.7%) had received nirsevimab prior to hospitalization, respectively (*p* = 0.8).

### 3.3. Independent Risk Factors for Severe Course and Characteristics of the IC Group

**Figure 2:**
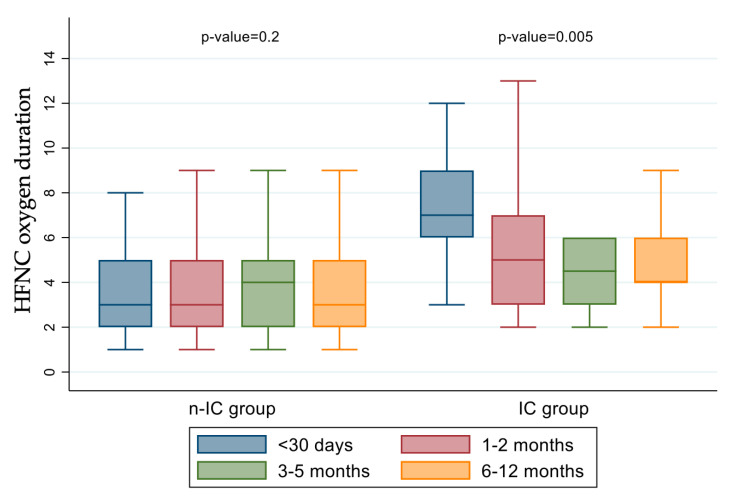
Correlation between duration of HFNC oxygen supplementation (days) and age according to disease severity.

The median duration of hospital stay in the IC group was 8 (IQr 5–11) days. The univariate and multivariate logistic analysis showed that younger age and having a comorbidity represented independent risk factors for a severe clinical course (Table 2). Furthermore, RSV infection, both isolated or combined with other respiratory viruses, correlated to a higher risk to belong to the IC group (Table 2). Season did not represent a risk factor for more severe disease. The duration of hospital stay in the IC group was similar between the two periods [S1: 8 (IQr 6–11); S2: 7 (IQr 5–9); *p* > 0.05]. When we compared the duration of oxygen supplementation and age within the two groups (n-IC and IC), we observed that there was no difference in the length of oxygen therapy according to the age-groups in the n-IC group, whereas a correlation was found in the IC group (*p* < 0.05) (Figure 2).

Thirty-one (27.9%) patients in the IC group were transferred to PICU or NICU because of worsening of clinical conditions, requiring invasive ventilation, and/or cardio-respiratory instability. In 13/31 (41.9%) HFNC was maintained, in 6 (19.3%) cases non-invasive mechanical ventilation was necessary, while 12 (38.7%) patients required invasive mechanical ventilation (IMV), after failing less intensive oxygen support. It is worth noting that none of the newborns admitted to NICU received IMV. Overall, IMV was used in only 1.1% of the studied population.

A total of 48 /111 (43.2%) patients of the IC group received parenteral dexmedetomidine with a median maximum dose of 1 μg/kg/h (range, 0.5–1.4 μg/kg/h) as the only continuously administered sedative agent in combination with non-invasive ventilatory support to facilitate support during bronchiolitis care.

## 4. Discussion

Bronchiolitis is responsible for a large disease burden in children, particularly in extremely young infants and those with underlying diseases. Currently, in Italy national insurance provides RSV immunoprophylaxis with nirsevimab, which has been widely demonstrated to be effective in protecting all infants from RSV-associated hospitalization, regardless of the presence of high-risk comorbidities [14,15,16].

We performed a large study evaluating the characteristics of patients with bronchiolitis requiring intensive care management across two subsequent seasons (pre- and post-nirsevimab implementation). In both analyzed seasons, the peak of bronchiolitis and RSV infection was observed in December–January, aligning more closely with the seasonality expected before the SARS-CoV-2 pandemic [17].

The number of admissions related to bronchiolitis decreased from about 630 patients in 2023–2024 to 420 in 2024–2025. A specific assessment of nirsevimab’s effectiveness in reducing hospitalizations was beyond the aims of our study and several confounders including changes in testing, care-seeking behavior and seasonal variations should be considered. Nevertheless, our findings support data from all the countries where nirsevimab immunization was introduced in 2023–2024 (e.g., USA, France, Spain, and Luxembourg) confirm its high effectiveness in reducing RSV-related hospitalizations [15,16,18,19]. Nevertheless, the proportion of severe infections requiring intensive management was approximately 10% in both seasons.

Campania region represents a peculiar example to study the effects of nirsevimab administration in the 2024–2025 season. First, patients eligible for prophylaxis were only those born after 1 August 2024, unlike other Italian Regions that included children born after April or even before. Second, nirsevimab administration was provided by general pediatricians or vaccination centers in case of infants born between 1 August and 10 November 2024, whereas it was proposed to parents in the Neonatology Units for infants born between 11 November 2024 and 14 April 2025 [10]. This local policy led to a high nirsevimab coverage of approximately 89% in infants born after 11 November 2024 [20]. Nevertheless, although official estimates regarding immunizations performed at vaccination centers or by general pediatricians are lacking and unpublished data are only partially available, a lower coverage, likely approaching 70%, was reported in infants born between 1 August and 11 November 2024. Such a gap was mainly due to organizational issues and to the delayed supply of monoclonal antibodies during the first months of the immunization campaign. Therefore, at the beginning of the RSV epidemic season, newborns and young infants who had received prophylaxis in their first days of life coexisted in the same weeks with older, non-immunized infants.

In addition, our study population also included cases of bronchiolitis unrelated to RSV infection. As our primary outcome was to assess the characteristics of patients with a severe course, rather than the efficacy of nirsevimab, this heterogeneity regarding the etiology of bronchiolitis allowed us to better investigate the risk factors associated with severe disease course.

Overall, about 10% of our patients developed a severe disease requiring admission to Sub-Intensive or Intensive Care Units. Recently, Bandeira et al. reported a variation in ICU admission rates, increasing from 13% in 2019 to 31% in 2023 [21]. Other studies reported increased ICU admissions of children with bronchiolitis during the post-COVID-19 season (2022–2023) compared to previous years [22]. However, the reasons for this increase are still unclear. Bronchiolitis in the first year after COVID-19 pandemic had peculiar features, with a peak of RSV infection in Italy occurring in November, in advance compared to pre-pandemic seasons [23]. The increased severity observed may have several explanations, including the complete removal of restrictions applied during the pandemic, and the “immune debt” consisting in an increased susceptibility to infections following a prolonged period of reduced exposure to pathogens that leads to a lower herd immunity. Nevertheless, a rise in coinfections and different attitudes towards the more aggressive treatment of respiratory distress after the pandemic era likely represent further contributing factors [21,22,23,24]. It is worth noting that, despite being admitted to ICU for respiratory support and strict monitoring, only 1% of infants belonging to the IC group required IMV. Ma et al. reported a proportion of severe RSV bronchiolitis between 2 and 4% from 2012 to 2018 [25]. However, RSV infection severity was defined based on the need for IMV support [25]. Such a definition may be misleading, as the use of IMV is generally reserved to a limited proportion of very severe cases, as it is often of poor benefit or even counterproductive in bronchiolitis. Our choice of considering inclusion criteria for the “severe course group” other than intubation only helped to better understand risk factors for more severe cases. Kirolos et al. analyzed risk factors for severe RSV-related bronchiolitis in 709 patients and documented a severe disease course in about 29% of cases during the pre-nirsevimab implementation era [26]. In this study, severe disease was more likely in infants under 6 months, with a history of preterm birth or with comorbidities. Our analysis was not limited to RSV-bronchiolitis only, but included all cases, including those with unknown etiology or not performed molecular tests. In line with previous findings, we observed that younger age, comorbidities, and RSV infection were independent risk factors for ICU admission. Interestingly, more than 80% of children in the IC-group were otherwise healthy infants, thus confirming that RSV preventive strategies should not be limited to high-risk groups, but should be extended to all infants.

Bronchiolitis can affect infants and children under 2 years of age, but it is most common in the first year of life, peaking between 3 and 6 months. In this age-range clinical severity is increased compared to older age [11]. Nevertheless, a consensus is missing regarding the definitive upper limit of age for its diagnosis. In our study, we selected only patients under 12 months of age in order to select children with a more likely clinical diagnosis, a less wide spectrum of symptoms, and a higher probability of hospitalization and severe course. Furthermore, we stratified children in four age groups rather than considering age as a continuous variable. Although somewhat arbitrary, this choice was driven by the consideration that expressing results according to age groups is more informative and clinically interpretable. Indeed, when considering age as a continuous variable, a linear effect per day increase is assumed, and this may not adequately reflect the non-linear and heterogeneous vulnerability to bronchiolitis throughout the first year of life, especially when comparing early neonatal with late infancy periods. Another consideration is related to the high variability of the criteria used to define severe disease [27]. Severe bronchiolitis is often used as an endpoint in clinical trials. The lack of agreement between definitions significantly limits the interpretation and comparison of the findings from different studies. In the present study, severity was defined based on the need for intensive monitoring and care, regardless of patient’s age and/or presence of comorbidities. Furthermore, inclusion in the IC-group was not only based on the need for IMV, which is rarely used in bronchiolitis, but also on the need for a sub-intensive management, that, in our hospital, allows the strict monitoring of vital parameters, HFNC oxygen therapy, and parenteral sedation. Such criteria to define severity were preferred to the kind of respiratory support used as most patients, even though admitted to IC units, received maximal HFNC oxygen supplementation, with only 12 children and no neonate treated with IMV. Therefore, the need for an intensive or sub-intensive setting of care was considered a more reliable surrogate of patients’ clinical severity. It is worth noting that since the Pediatric Emergency Department of Santobono-Pausilipon Children’s Hospital is the reference facility for pediatric Sub-Intensive Care Unit and ICU in Campania Region, it is common for children to be referred from other hospitals to our center, allowing us not to lose records. A more standardized clinical definition of bronchiolitis, as well as the use of a validated clinical severity score, would allow a more accurate comparison between studies with possible useful implications on patients’ management.

Our study has both strengths and limitations. In addition to the large population enrolled and to its prospective and multicenter design, an undeniable strength is the inclusion of all infants hospitalized because of bronchiolitis and the availability of etiological diagnoses for most patients. Furthermore, one of the major limitations of the available studies is the collection of data obtained from administrative databases that might lead to the inclusion of RSV-negative cases but also to the exclusion of RSV-positive subjects classified elsewhere [21]. In addition, comorbidities may also be lacking in studies based on administrative data because of coding errors or missing information. To avoid these issues, in our population, data were collected by reviewing medical charts and not by extraction based on the International Classification of Diseases (ICD) codes. This allowed us to retrieve individual data and better analyze the relationship between risk factors and poor outcomes. On the other hand, our study is limited by the lack of some data, e.g., parents’ education, household smoking, or vaccination coverage rates. Furthermore, an extensive respiratory panel was not available for all included patients. Unfortunately, such diagnostic devices were not available during the entire study period, and, when necessary, short panels were used as an alternative. Although the choice to perform extensive or short viral panels was mainly driven by the availability of extensive PCR-based panels at laboratory sites, we found that severe patients underwent extensive panels more frequently, suggesting a more intense diagnostic effort. Nevertheless, this bias did not entail a higher proportion of coinfections in IC patients. Rapid antigen test for RSV was used only in approximately 5% of the study population, due to unavailability of PCR-based tests. Despite different sensitivities between these diagnostic techniques, the small proportion of patients undergoing antigenic tests unlikely affected our findings. The analysis of the role of coinfection was, therefore, investigated only in about half of cases. However, the high number of enrolled patients demonstrates the significant role of RSV infection, more than coinfections, in determining a severe disease course.

## 5. Conclusions

Severe bronchiolitis requiring intensive care management accounts for about 10% of cases, with IMV needed in only 1% of cases. Age, comorbidities, and RSV positivity represented independent risk factors for a severe disease course. Nirsevimab administration, despite an optimal coverage rate, was not achieved in our region during the 2024–2025 period because of organizational issues, determined a reduction in the overall number of hospitalizations and in the absolute number of patients with severe bronchiolitis. Nevertheless, no significant difference in the rate of patients experiencing a severe disease course was observed between the pre- and post-nirsevimab seasons. This demonstrates that, despite nirsevimab implementation, the careful assessment and monitoring of patients with bronchiolitis is still needed for the early identification of patients at risk of severe disease.

## Figures and Tables

**Table 1:** Baseline patients’ characteristics by disease severity.

		Intensive Care		
	Total ^1^	n-IC Group (n = 945)	IC Group (n = 111)	*p*
Season (n, %)				
2023/2024	633 (59.9)	564 (59.7)	69 (62.2)	
2024/2025	423 (40.1)	381 (40.3)	42 (37.8)	0.614
Age group (n, %)				
<30 days	150 (14.2)	120 (12.7)	30 (27)	
1–2 months	500 (47.3)	446 (47.2)	54 (48.7)	
3–5 months	245 (23.2)	227 (24)	18 (16.2)	
6–12 months	161 (15.2)	152 (16.1)	9 (8.1)	<0.001
Gender (n, %)				
Female	475 (45)	417 (44.1)	58 (52.3)	
Male	581 (55)	528 (55.9)	53 (47.8)	0.104
Preterm birth (n, %)				
No	734 (69.5)	665 (86.8)	69 (80.2)	
Yes	118 (11.2)	101 (13.2)	17 (19.8)	0.904
Comorbidities (n, %)				
No	866 (82)	777 (92.6)	89 (80.9)	
Yes	83 (7.9)	62 (7.4)	21 (19.1)	<0.001
RSV ^2^ (n, %)				
No	275 (26)	261 (28.1)	14 (12.6)	
Yes	765 (72.4)	668 (71.9)	97 (87.4)	<0.001
Coinfections ^3^ (n, %)				
No	379 (35.9)	328 (69)	51 (66.2)	
Yes	173 (16.4)	147 (31)	26 (33.8)	0.621
CRP ^4^ > 10 mg/dL (n, %)				
No	735 (69.6)	674 (77.6)	61 (55.5)	
Yes	244 (23.1)	195 (22.4)	49 (44.6)	<0.001
Oxygen therapy (n, %)				
No	410 (38.8)	405 (42.9)	5 (4.5)	
Yes	646 (61.2)	540 (57.1)	106 (95.5)	<0.001
Antibiotic therapy (n, %)				
No	685 (64.9)	657 (69.7)	28 (25.2)	
Yes	369 (34.9)	286 (30.3)	83 (74.8)	<0.001
Inhaled steroids (n, %)				
No	306 (29)	283 (30)	23 (20.7)	
Yes	747 (70.7)	659 (70)	88 (79.3)	0.041
Oral steroids (n, %)				
No	718 (68)	629 (66.5)	89 (80.2)	
Yes	338 (32)	316 (33.6)	22 (19.8)	0.003
Parenteral steroids (n, %)				
No	842 (80)	799 (84.7)	43 (39.1)	
Yes	211 (20)	144 (15.3)	67 (60.9)	<0.001
Inhaled adrenaline (n, %)				
No	740 (70.3)	685 (72.6)	55 (50)	
Yes	313 (29.7)	258 (27.4)	55 (50)	<0.001
Chest X ray consolidation (n, %)				
No	285 (67.2)	241 (71.9)	44 (49.4)	
Yes	139 (32.8)	94 (28.1)	45 (50.6)	<0.001
Hospital stay (days) *	4 (3–6)	4 (2–6)	8 (5–11)	<0.001
HFNC ^5^ oxygen therapy (days) *	4 (2–6)	3 (2–5)	6 (4–8)	<0.001

^1^ Due to missing data, for some parameters the total may be lower than 1056. ^2^ Respiratory syncytial virus. ^3^ Data available only for patients who underwent complete respiratory panel. ^4^ C-reactive protein. ^5^ High-flow nasal cannula. * Median and interquartile ranges.

**Table 2:** Risk factors for a severe course in patients with bronchiolitis.

	Univariate Analysis				Multivariate Analysis			
	OR ^1^	95% CI		*p*	OR	95% CI		*p*
Age group								
<30 days								
1–2 months	0.48	0.30	0.79	<0.0001	0.37	0.22	0.64	<0.0001
3–5 months	0.32	0.17	0.59	<0.0001	0.27	0.14	0.52	<0.0001
6–12 months	0.24	0.11	0.52	<0.0001	0.20	0.09	0.46	<0.0001
Comorbidities	2.96	1.72	5.08	<0.0001	3.54	1.97	6.35	<0.0001
RSV ^2^	2.71	1.52	4.83	<0.0001	3.30	1.77	6.13	<0.0001
CRP ^3^ > 10 mg/dL	2.78	1.85	4.18	<0.0001	2.61	1.70	4.01	<0.0001
Season	0.90	0.60	1.35	0.61	0.77	0.50	1.19	0.25

^1^ Odds ratio. ^2^ Respiratory syncytial virus. ^3^ C-reactive protein.

## Data Availability

The original contributions presented in this study are included in the article. Further inquiries can be directed to the corresponding author.

## Abbreviations

The following abbreviations are used in this manuscript:

PICU	Pediatric Intensive Care Unit
NICU	Neonatal Intensive Care Unit
SpO_2_	Peripheral Oxygen Saturation
IC	Intensive Care
RSV	Respiratory Syncytial Virus
CRP	C-Reactive Protein
OR	Odds Ratio
HFNC	High-Flow Nasal Cannula
IMV	Invasive Mechanical Ventilation
